# Solitary Fibrous Tumor of the Postcricoid Region: A Case Report and Literature Review

**DOI:** 10.1155/2013/908327

**Published:** 2013-08-20

**Authors:** Brian Cervenka, Brenda Villegas, Uttam Sinha

**Affiliations:** ^1^Department of Otolaryngology-Head and Neck Surgery, Keck School of Medicine of USC, 1200 North State Street, Room 4136, Los Angeles, CA 90033, USA; ^2^Department of Otolaryngology-Head and Neck Surgery, University of California, Davis, 2521 Stockton Boulevard, Suite 7200, Sacramento, CA 95817, USA

## Abstract

Solitary fibrous tumor (SFT) is a rare mesenchymal neoplasm that can present essentially anywhere in the body. Presentations in the hypopharynx are extremely rare with only two previous cases reported. We report the first case of postcricoid SFT occurring in a 58-year-old male requiring a microsuspension laryngoscopy excision following an unsuccessful transoral robotic attempt. The excision was uneventful, and the patient is currently without recurrence. Current management strategies of the hypopharyngeal SFT, the unique differential diagnosis, and challenges in surgical approaches in the postcricoid region are discussed.

## 1. Introduction 

Solitary fibrous tumor (SFT) is a rare spindle cell, mesenchymal neoplasm characterized by the proliferation of thin-walled vessels and collagen producing cells [1]. SFT has been described in almost every organ in the human body, but presentation in the hypopharynx is extremely rare, with only two previously reported cases [2, 3]. We present the first case of SFT ever reported in the English literature originating from the postcricoid region of the hypopharynx. A literature review of current management strategies of the hypopharyngeal SFT, the unique differential diagnosis, and challenges in surgical approaches in the postcricoid region is then presented.

## 2. Case Report 

**Figure 1:**
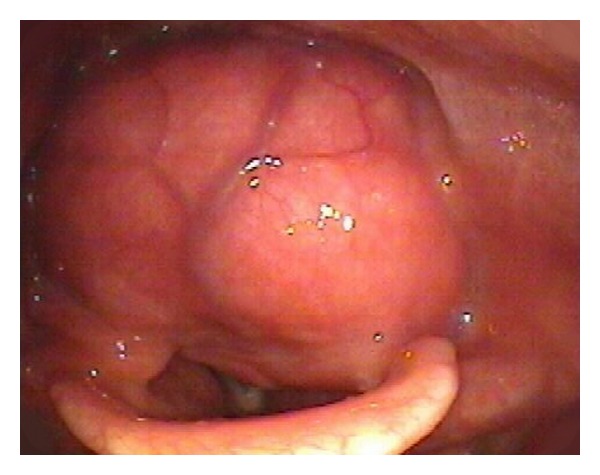
Video stroboscopy image of the postcricoid SFT demonstrating a large soft tissue mass in the postcricoid region encroaching the laryngeal inlet.

The patient is a 58-year-old African-American male with a past medical history of hypertension, hypothyroidism, and asthma, who initially presented to an otolaryngologist with complaints of one-year history of intermittent tightness in the throat, progressive shortness of breath, dysphagia, and dysphonia. The patient denied any history of tobacco or alcohol use. The patient was referred to USC Department of Otolaryngology in October 2010. Clinical evaluation was unremarkable. There was no neck mass or lymphadenopathy. Video stroboscopy demonstrated a large mass in the postcricoid region encroaching the laryngeal inlet. The left vocal fold appeared to be immobile (Figure 1).

**Figure 2:**
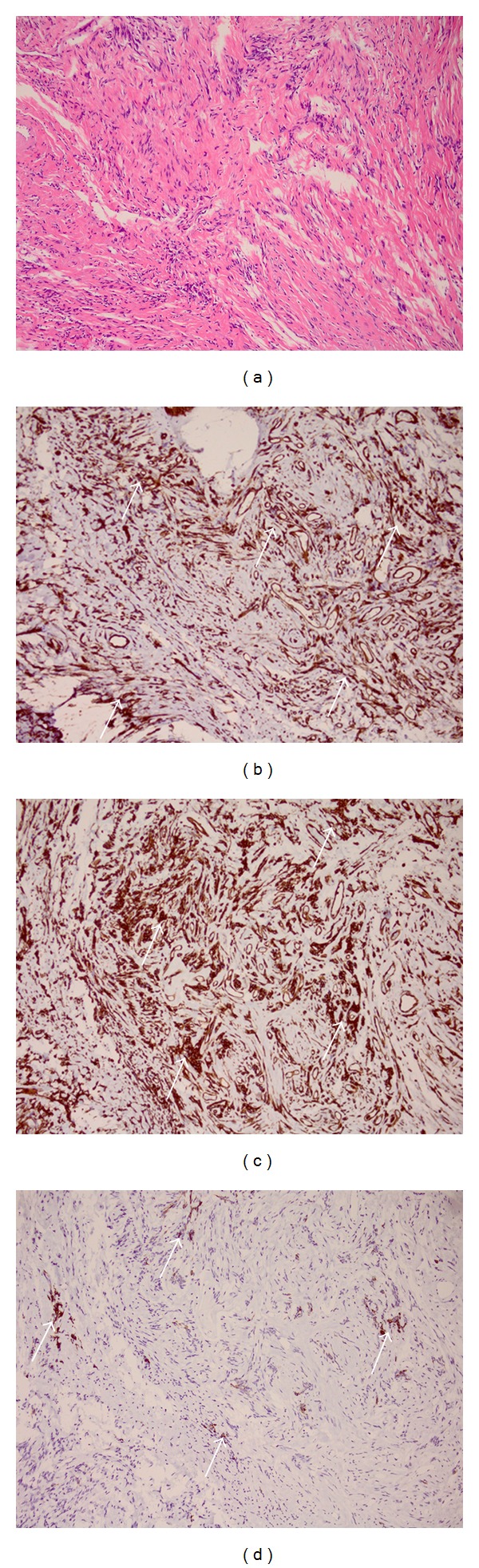
Histological examination of the postcricoid SFT (magnification in all images 100x). (a) Abundant spindle-shaped tumor cells with characteristic whorled arrangement (hematoxylin and eosin staining); (b) strong CD34 immunoreactivity (arrows); (c) strong vimentin immunoreactivity (arrows); (d) focal and weak desmin immunoreactivity (arrows).

Computerized tomography scan demonstrated a heterogeneous, diffusely enhancing mass of the postcricoid region extending to the left pyriform sinus. It measured 4.1 cm right to left and 3.6 cm anteroposteriorly, and it displaced the left thyroid cartilage and arytenoid cartilage a few millimeters anteriorly. There was adjacent thickening of the left aryepiglottic fold. Direct laryngoscopy and biopsy were performed in November 2010. A mucosally covered, partially mobile mass in the postcricoid area that prolapsed into the esophageal inlet was noted. Permanent pathology examination of the 0.5 cm × 0.4 cm × 0.4 cm biopsy specimen demonstrated a spindle-shaped cellular neoplasm staining positive for CD34 and vimentin and focally for desmin (Figures 2(a)–2(d)). 

The mass was negative for pan keratin, S100, and CD117. Based on the low mitotic rate and expression of MIB-1, the lesion was classified as having low malignant potential.

In December 2010, the patient was scheduled to undergo transoral robotic excision of the postcricoid SFT. The required field visualization was not possible using the transoral robotic mouth gag. A direct microsuspension laryngoscopy approach using 500 mm lens and a fiber optic laser set at 12 watts was utilized. A midline, vertical incision through the mucosa superficial to the mass was made and extended from the posterior commissure to the esophageal inlet. The mucosa was then separated off the mass by using blunt dissection with laparoscopic scissors. Once the mass was completely exposed, it was completely resected with laparoscopic scissors. Hemostasis was obtained, and redundant mucosa from the postcricoid region was removed. The patient was kept intubated overnight and extubated uneventfully next day. He was discharged from the hospital on the same day after confirming safe oral intake. Pathology examination showed 5 cm × 5 cm × 3 cm pink-tan, lobulated, mass and confirmed preoperative diagnosis of SFT.

**Figure 3:**
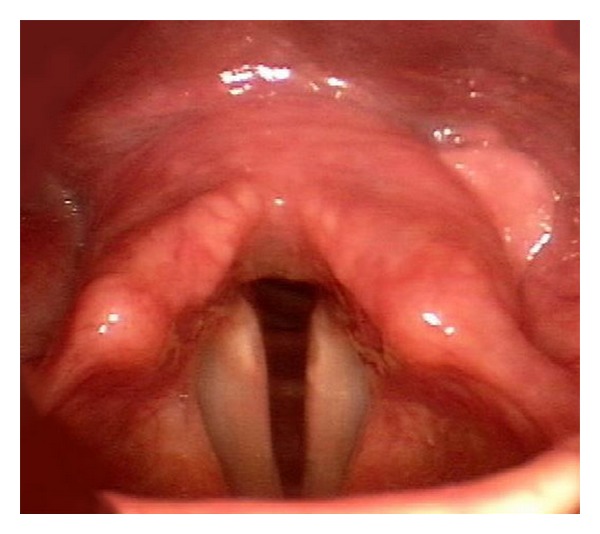
Video stroboscopy image of the postcricoid region 22 months after microexcision of SFT demonstrating no recurrence of disease.

Currently, it is 22 months after-excision and the patient reports significant improvement of his breathing, voice, and swallowing. The last video stroboscopy examination performed in June 2012 demonstrated no recurrence of disease (Figure 3).

## 3. Discussion 

Solitary fibrous tumor (SFT) was first described by Klemperer and Coleman in 1992 [4]. Initially thought to originate from mesothelial cells and submesothelial fibroblasts, it was referred to as localized fibrous mesothelioma, solitary fibrous mesothelioma, or submesothelial fibroma [5]. Subsequent studies, utilizing tissue culture and immunohistochemistry, demonstrated that these neoplasms are actually of mesenchymal origin, which explained the increasing body of literature demonstrating extrathoracic primary locations [6]. Previously thought to occur most commonly in the pleura, this notion has recently been challenged as it continues to be reported in extrapleural sites [7]. Currently, SFT has been described in almost every organ in the human body. It has been described in the head and neck regions, including the nose and paranasal sinuses, nasopharynx, major salivary glands, larynx, thyroid gland, skin, deep soft tissue, oral cavity, parapharyngeal space, and orbit [1, 5]. 

Two authors have previously reported hypopharyngeal SFTs, but both originated from the pyriform sinus [2, 3]. All three cases (including the present case) of SFT of the hypopharynx occurred in men. The ages at diagnosis were between 32 and 58, and the average was 45. Presenting symptoms were related to the mass effect of the tumor and included dysphonia, dyspnea, a feeling of tightness in the chest, dysphagia, and weight loss [2, 3]. SFT in other locations has been demonstrated to cause hypoglycemia secondary to IGF-2 production, but this has not been demonstrated in the hypopharynx [8]. Airway compromise is a concern if in close proximity to the airway. Our patient presented with progressive shortness of breath leading him to seek treatment, and the mass could be seen encroaching on the laryngeal inlet. Mussak et al. reported that their patient, although not reporting dyspnea, had a near occlusion of the airway by the hypopharyngeal mass [2]. This has also been described in cases presenting in the larynx. Thompson et al. described a SFT of the true vocal cord causing what the patient thought was exacerbation of asthma symptoms and led to severe respiratory distress requiring an urgent tracheostomy and subsequent excision [1]. Because of the paucity of cases, there are no established risk factors for SFT occurring in the larynx or hypopharynx. Both Hanna et al.'s patient and the patient described in our case denied alcohol and tobacco use [3]. Only two of the eight recorded cases presenting in the larynx had a smoking history, and no other common etiologic factors were determined [1].

SFT most commonly is a benign neoplasm. Eighty-seven percent of reported SFT, as of 2005, exhibited benign clinical behavior [9]. Malignancy can occur and is associated with histologic features of marked hypercellularity and pleomorphism, infiltrative borders, necrosis and greater than four mitoses per 10 high-power field [8]. Large tumor size is also associated with a more aggressive behavior [8]. Neoplasms located in the pleura, mediastinum, abdomen, pelvis and retroperitoneum tend to behave more aggressively [10]. Metastasis has been described to the lungs, bone, and liver but is very rare [10]. In the hypopharynx, there have been two reported benign cases and one histologically malignant case that was not associated with any local or distant invasion [2, 3]. 

The preferred treatment of SFT is complete excision. Negative margins are essential as positive margins have been associated with a higher recurrence rate in extralaryngeal cases [8]. Because it is a vascular lesion, CO_2_ lasers are preferred, although care must be taken not to obscure margin visualization with excessive cauterization [1, 11]. Cervical lymph node dissection is not indicated [11]. Radiation and chemotherapy have not been used [8]. Although SFT is generally benign, close followup is recommended because of the unpredictable metastatic and recurrence behaviors reported in other locations [9]. Of the two SFTs previously reported in the hypopharynx, one was treated with a transcervical open excision and the other with an endoscopic excision [2, 3]. Mussak et al. reported a SFT originating from the lateral hypopharyngeal wall and measuring 4.5 cm × 4.0 cm × 3.2 cm that was excised through an open transcervical approach [2]. The case described by Hanna et al. originated in the right pyriform sinus was 4.1 cm × 3.0 cm × 1.2 cm and was resected endoscopically in multiple segments [3]. The present case originated from the postcricoid region and was a 5 cm × 5 cm × 3 cm lesion. A transoral robotic approach was attempted but was not possible because of  insufficient field visualization. Despite the large size of the SFT (the largest ever reported in the hypopharynx), we were able to completely excise the neoplasm endoscopically. 

The differential diagnosis of postcricoid masses is the same as in the other divisions of the hypopharynx and includes, most commonly, squamous cell carcinoma (SCC) and other less common entities such as adenocarcinomas, lymphomas, and sarcomas [12]. Plummer-Vinson is a syndrome of esophageal webs, iron deficiency anemia, and dysphagia specifically associated with SCC presenting in the postcricoid region [13]. The postcricoid region presents a unique surgical challenge because it is positioned between two rigid structures, the posterior surface of the cricoid cartilage and C5 and C6 vertebral bodies. This reduces the flexibility required to enhance visualization when approaching from a transoral route. Additionally, the current microexcision instruments available have limitations in working in this area.

SFT is a difficult lesion to diagnose based on clinical and histological findings alone, making immunohistochemistry essential complimentary assay. SFT is characteristically immunoreactive for CD34, CD99, bcl-2, and vimentin and typically negative for cytokeratin, s100, SMA, and desmin [8]. Strong CD34 immunoreactivity of SFT is especially important in differentiating the neoplasm from hemangiopericytoma, which does not stain as consistently or intensely for CD34 [14]. In the larynx, the combination of CD34 and bcl-2 positivity has consistently been associated with SFT [2]. The hypopharyngeal case described by Mussak et al. was positive for CD34, CD99, bcl-2, and S100 [2]. Hanna et al. described a hypopharyngeal SFT positive for CD34 and vimentin [3]. The current case is unique, as in addition to strong CD34 and vimentin staining, it stained weakly and focally for desmin, which is very rare in any location and has never been reported in the hypopharynx or larynx. 

SFT is a rare mesenchymal neoplasm that can present essentially anywhere in the body. There is an increasing body of evidence describing extrathoracic presentations. Presentations in the hypopharynx are extremely rare with only two previous cases ever reported, and no cases have ever been reported originating from the postcricoid region, as was seen in this report. These neoplasms are generally benign, and the treatment is complete excision. Transoral CO_2_ laser approaches have recently gained popularity for excision. The postcricoid region presents a unique surgical challenge because of minimal working space and flexibility. Immunohistochemistry is essential for making the diagnosis of SFT. CD34, CD99, Bcl-2, and vimentin are characteristically positive. Our case demonstrated patchy desmin staining, which is rare and has never been reported in the hypopharynx or larynx. 

## References

[1] Thompson LDR, Karamurzin Y, Wu ML, Kim JH (2008). Solitary fibrous tumor of the larynx. *Head and Neck Pathology*.

[2] Mussak EN, Tu JJ, Voigt EP (2005). Malignant solitary fibrous tumor of the hypopharynx with dysphagia. *Otolaryngology—Head and Neck Surgery*.

[3] Hanna GJ, Grant N, Wycherly B (2011). Pathology quiz case 2. Solitary fibrous tumor (SFT) of the hypopharynx. *Archives of Otolaryngology—Head and Neck Surgery*.

[4] Klemperer P, Coleman BR (1992). Primary neoplasms of the pleura. A report of five cases. *The American Journal of Industrial Medicine*.

[5] Brunnemann RB, Ro JY, Ordonez NG, Mooney J, El-Naggar AK, Ayala AG (1999). Extrapleural solitary fibrous tumor: a clinicopathologic study of 24 cases. *Modern Pathology*.

[6] Morgan MB, Smoller BR (2000). Solitary fibrous tumors are immunophenotypically distinct from mesothelioma(s). *Journal of Cutaneous Pathology*.

[7] de Saint Aubain Somerhausen N, Rubin BP, Fletcher CDM (1999). Myxoid solitary fibrous tumor: a study of seven cases with emphasis on differential diagnosis. *Modern Pathology*.

[8] Gold JS, Antonescu CR, Hajdu C (2002). Clinicopathologic correlates of solitary fibrous tumors. *Cancer*.

[9] Sandvliet RH, Heysteeg M, Paul MA (2000). A large thoracic mass in a 57-year-old patient. *Chest*.

[10] Fletcher CDM, Unni KK, Mertens F (2002). *Pathology and Genetics of Tumours of Soft Tissue and Bone*.

[11] Dotto JE, Ahrens W, Lesnik DJ, Kowalski D, Sasaki C, Flynn S (2006). Solitary fibrous tumor of the larynx. A case report and review of the literature. *Archives of Pathology and Laboratory Medicine*.

[12] Flint PW, Haughley BH, Lund VJ (2010). *Cummings Otolaryngology-Head and Neck Surgery*.

[13] Larsson LG, Sandström A, Westling P (1975). Relationship of Plummer-Vinson disease to cancer of the upper alimentary tract in Sweden. *Cancer Research*.

[14] Alobid I, Alós L, Maldonado M, Menéndez LM, Bernal-Sprekelsen M (2005). Laryngeal solitary fibrous tumor treated with CO_2_ laser excision: case report. *European Archives of Oto-Rhino-Laryngology*.

